# X chromosome effects and their interactions with mitochondrial effects

**DOI:** 10.1186/1471-2156-6-S1-S157

**Published:** 2005-12-30

**Authors:** Jack W Kent, Loren R Lease, Michael C Mahaney, Thomas D Dyer, Laura Almasy, John Blangero

**Affiliations:** 1 Department of Genetics, Southwest Foundation for Biomedical Research, San Antonio, TX 78245, USA

## Abstract

We report a simple and rapid method for detecting additive genetic variance due to X-linked loci in the absence of marker data for this chromosome. We examined the interaction of this method with an established method for detecting mitochondrial linkage (another source of sex-asymmetric genetic covariance). When applied to data from the Collaborative Study on the Genetics of Alcoholism, this method found evidence of X-chromosomal linkage for one continuous trait (ntth1) and one discrete trait (SPENT). Evidence of mitochondrial contribution was found for one discrete trait (CRAVING) and three continuous traits (ln(CIGPKYR), ecb21, and tth1). Results for ntth1 suggest that methods that do not also allow for male-female heterogeneity in environmental variance may be overly conservative in detection of X-chromosomal effects.

## Background

Although the X chromosome is one of the largest in the human genome, genetic analysis software packages have been slow to incorporate routines for X-linked loci. This gap is closing: experimental two-point X-linkage analysis routines are now available in MERLIN [1] and SOLAR [2], and Ekstrøm [3] recently proposed an algorithm for multipoint linkage analysis of the X. Even prior to localizing quantitative trait loci within the X, however, it may be useful to determine the additive genetic effect of the X *as a whole*, both to allow the investigator to decide whether to pursue a more detailed analysis of the chromosome, and to improve the specificity of variance components models for autosomal linkage.

In Ekstrøm [3], in part to avoid assumptions about presence or absence of dosage compensation, both the linkage model and the model for the residual additive genetic effect of the X are estimated in terms of separate variance components for male-male, female-female, and male-female relative pairs. Here we offer a simplified mechanism for analyzing the 'X effect' as a single parameter (for ease of both computation and interpretation), even when marker data for this chromosome are unavailable. Our formulation derives from Bulmer's [4] dosage compensation model, which is applicable to the majority of X-chromosomal loci in humans and other eutherian mammals. Direct comparison of our simplified approach with that of Ekstrøm [3] is beyond the scope of this paper. However, based on a simulation study that does make this comparison by Kent et al. [5], we believe that the simplified method has equal or greater power to detect dosage compensated loci, and is conservative for non-lyonizing loci.

We used the method as a tool address the more general question of the interaction of sex-related effects in complex traits. Both X-linkage and mitochondrial linkage display asymmetric patterns of allele transmission from males and females, and either may be confounded with genotype × sex or environment × sex interaction effects on traits influenced by autosomal genes. A fully accurate specification of the variance components model for inheritance of a particular trait should distinguish among these possibilities. A companion study [6] examines possible mitochondrial contributions to a set of alcoholism-related phenotypes from the Collaborative Study on the Genetics of Alcoholism (COGA); here we extend this investigation to X-chromosomal effects.

## Methods

### The COGA data

We used behavioral and electrophysiological phenotypes in the subset of COGA data generously provided for the Genetic Analysis Workshop 14. The data collection process, including acquisition of written informed consent from all participants and approvals by Institutional Review Boards, are described more fully elsewhere [7].

### Effect of the X chromosome

We have implemented an extension to the genetic analysis package SOLAR [2] to estimate the additive genetic effect of X-linked loci. For humans and many other placental mammals in which most X-linked loci are subject to random inactivation in females, it should be a reasonable first approximation to employ a simple dosage-compensation model [4]. The additive contribution of an X-linked locus is simply the effect of the single allele in the male, or the single active allele in a homozygous female. As an approximation, the effect of the locus in heterozygous females is just the average of the allele effects, because each female is a mosaic of cells in which one or the other X is inactivated at random. Under these assumptions, the mean X effect is the same in both sexes, while the variance due to the X in males is twice that in females. This linear relationship between the variances allows us to express the additive effect of the X in one sex in terms of the other. For example, in terms of the female variance σ^2^_Xf_,

**Ω **= 2**Φ**σ^2^_g _+ 2**Ψ**σ^2^_Xf _+ **I**_f_σ^2^_ef _+ **I**_m_σ^2^_em_,     (1)

where **Ω** is the matrix of phenotypic correlations between pairs of individuals,** Φ** is the matrix of *autosomal *kinship coefficients, and **Ψ** (using the notation of Ekstrøm [3]) is a new matrix of X-chromosome-specific coefficients of relationship for male-male, female-female, and male-female pairs (Table 1). Like the elements of **Φ**, the elements of **Ψ** can be calculated recursively from pedigree data, as by the algorithm in MINX (MERLIN in X [1,8]).

Inclusion of sex-specific identity matrices and random environmental variances (**I**_f_σ^2^_ef _and **I**_m_σ^2^_em_) in Equation 1 is desirable because the different σ^2^_Xf _and σ^2^_Xm _may appear as different environmental variances in the restricted model where σ^2^_Xf _= σ^2^_Xm _= 0, and also to account for genotype × sex or environment × sex interactions, if any. An important exception is that discrete traits (e.g., CRAVING) are analyzed using heritability parameterization with the phenotypic variance in both sexes fixed at 1 (because the variance-components model for discrete traits assumes an underlying liability with distribution *N*(0, 1)).

The significance test is straightforward, testing the ratio of the likelihood of the model with the single added parameter σ^2^_Xf _to that of the restricted model. Because σ^2^_Xf _is tested on its lower bound, the likelihood ratio statistic is distributed as a 1/2:1/2 mixture of χ^2 ^with one degree of freedom, and a point mass at zero [9].

### Mitochondrial effect

We used the mitochondrial identity-by-descent (IBD) calculation implemented in SOLAR [10] (and see discussion in Lease et al. [6]). The additive genetic variance due to the mitochondrion can be added to the basic polygenic model as

**Ω **= 2**Φ**σ^2^_g _+ **M**σ^2^_mt _+ **I**_f_σ^2^_ef _+ **I**_m_σ^2^_em_,     (2)

where **M** is the mitochondrial IBD matrix. For comparison to the X effects, we retain separate environmental variance terms for males and females (except for the discrete traits). The significance of the model that includes the mitochondrial variance is tested against the restricted model (σ^2^_mt _= 0) by a likelihood ratio test.

## Results and Discussion

In the following, evidence of mitochondrial or X effect is listed as significant at *p *< 0.05, without correction for multiple testing (the traits are genetically and phenotypically correlated [6] and measured in the same individuals). Note, however, that a modified Bonferroni correction that accommodates these genetic correlations, described by Lease et al. [6], yields a more stringent experiment wise type I error rate = 0.01.

### Mitochondrial and X effects in discrete traits

Phenotypes include 12 discrete behavioral traits related to alcohol consumption [7]. Two traits (BINGE and MORNING) did not show significant heritability in the polygenic model and were not considered further. Of the remaining traits (SMOKER, DESIRE, CRAVING, SPENT, NARROW, GAVEUP, BLACKOUT, WITHDRAWAL, HEALTH, and PSYCHO), data are shown only for those whose model likelihoods were increased by the addition of mitochondrial or X-effect terms (DESIRE, which showed a marginally significant X effect, is also shown). The sequence of analysis was: estimation of a polygenic model (POL); addition of a mitochondrial heritability term (MIT); addition of a heritability term for the additive genetic effect of the X (XFX). At each step the more restricted model was retained unless the model with the added parameter had a likelihood that was significantly greater at *p *< 0.05. Table 2 reports the *p*-values for the successive models and point estimates of the parameters retained in the final model for each trait. Trait SPENT showed a significant effect (*p *= 0.034) of the X chromosome, but the size of this effect (6% of total variance) is small. CRAVING showed a significant mitochondrial effect (*p *= 0.005), as reported [6], but no significant effect of the X chromosome. In each case the new parameter accounts for the entire additive genetic effect (i.e., h^2^_g _= 0) in the final model.

### Mitochondrial and X effects in continuous traits

We examined two behavioral traits, MAXDRINKS and CIGPKYR. Both measures were leptokurtic and were log_e_-transformed: κ before(after) transformation: MAXDRINKS 9.18 (0.06); CIGPKYR 9.69 (-1.46). We also examined 13 electrophysiology traits (ecb21, ntth1-4, ttdt1-4, ttth1-4). All showed a significant additive genetic component; data are shown only for those traits whose model likelihoods were increased by addition of more parameters. The sequence of analysis was: estimation of a (model POL); estimation of a polygenic model with separate environmental variance terms for the two sexes (EMF); addition of a mitochondrial variance term (MIT); addition of a variance term for the additive genetic effect of the X (XFX). At each step the more restricted model was retained unless the model with the added parameter had a significantly greater likelihood at *p *< 0.05. Table 3 reports the *p*-values for the successive models and the estimates of the parameters retained in the final model for each trait. Two traits (LNMXDK and ttdt2) had significantly different environmental variances in males and females, although neither showed significant mitochondrial or X effects. Three traits (LNCIGP, ecb21, and ttth1) showed evidence for mitochondrial effect. One trait (ntth1) showed a significant effect (*p *= 0.021) of the X.

### Interaction of sex-related effects

The initial screen of traits followed a step-wise procedure in which each additional parameter was retained in subsequent analyses only if it significantly increased the likelihood of the model. We were curious if estimating other combinations of parameters might alter the conclusions from this analysis. Table 4 presents results from a series of nested models for continuous traits LNCIGP and ntth1. (In the following, models A, B, etc. for each trait refer to their listing in Table 4.)

LNCIGP showed a significant mitochondrial effect in the initial screen (Table 3). This result is robust; the effect is detectable whether male and female environmental variances are constrained to be the same (model D vs. A, *p *= 0.037) or allowed to diverge (F vs. B, *p *= 0.030). In model E (σ^2^_mt _= 0) most of the variance actually due to the mitochondrion appears as residual additive genetic variance, with only minor misallocation of variance to the X.

A different pattern appears for ntth1, which showed a significant X effect in the initial screen. Interestingly, a significant X effect is detectable only if male and female environmental variances are allowed to differ (model E vs. B, *p *= 0.02): the addition of σ^2^_Xf _to the polygenic model barely increases the model likelihood (model H vs. A, *p *= 0.44). Different estimates of male and female environmental variance are retained in models that include the X effect, which may suggest genotype × sex interactions. Exclusion of σ^2^_Xf _does not yield a falsely significant estimate of σ^2^_mt _(model F vs. model B, *p *= 0.06).

## Conclusion

In this study we have applied a new method for detecting additive genetic variance due to the X chromosome to a representative set of real data. (Simulation studies to validate the method will be reported elsewhere.) We find that real mitochondrial effects (estimated by an established method) are not confounded with X effects. However, it appears that a true X effect can appear as heterogeneous environmental variance in males and females; indeed, in one case (ntth1), detection of an X effect *depended *on modelling this heterogeneity. This suggests that tests of X-chromosomal effect that constrain males and females to have equal environmental variances (as, for example, analyses of discrete traits) may be overly conservative.

## Abbreviations

COGA: Collaborative Study on the Genetics of Alcoholism

EMF: Model estimating separate environmental variances by sex

IBD: Identity-by-descent

MIT: Model estimating mitochondrial variance component

POL: polygenic model

XFX: Model estimating X-linked variance component

## Authors' contributions

JWK, TDD, and JB developed the methodology for analyzing the effect of the X chromosome. JWK conceived the study, performed the X-effect analyses, and drafted the manuscript. LRL and MCM performed the mitochondrial analyses. MCM and LA provided critical reviews of the methodology. TDD and LA coordinated preparation of the COGA data for investigators at SFBR. All authors have read and approved this manuscript.

## Figures and Tables

**Table 1:** Expected covariances for X-linked traits^a^

Relationship type	General^b,c^	Dosage compensation
Male-male	P_1_σ^2^_Xm_	2P_1_σ^2^_Xf_
Female-female	2φσ^2^_Xf_	2φσ^2^_Xf_
Male-female	(1/√2)P_1_σ_Xm_σ_Xf_	P_1_σ^2^_Xf_

^a^Expected additive genetic covariances due to the X chromosome without and with assumption of dosage compensation [4].

^b^P_1 _and P_2_, the respective probabilities that one and two alleles are shared IBD.

^c^φ, the autosomal (i.e., diploid) kinship coefficient = P_1_/4 + P_2_/2.

**Table 2:** Heritability estimates for selected discrete phenotypes

		Model test	*p*-Value
DESIRE		POL vs. sporadic	3.49 × 10^-6c^
		MIT vs. POL	0.067
		XFX vs. POL	0.050
final model:^a^	h^2^_g_: 0.48(0.12) + e^2^: 0.52(0.12)		
CRAVING		POL vs. sporadic	0.0003^c^
		MIT vs. POL	0.005^c^
		XFX vs. MIT	0.229
final model:^a^	h^2^_g_: 0.00(0.14^b^) + e^2^: 0.81(0.05) + h^2^_mt_: 0.19(0.05)		
SPENT		POL vs. sporadic	0.020^d^
		MIT vs. POL	0.356
		XFX vs. POL	0.034^d^
final model:^a^	h^2^_g_: 0.00(0.05^b^) + e^2^: 0.94(0.02) + h^2^_Xf_: 0.06(0.02)		

^a^Estimates (SE) of parameters retained in the final model (the final estimate of h^2^_g _is given even when = 0). h^2^_g_, autosomal additive genetic heritability; h^2^_mt_, heritability due to the mitochondrion; h^2^_Xf_, X-linked heritability in females; e^2^, environmental component.

^b^One-tailed empirical SEs estimated for point estimates at the boundary.

^c^*p *< 0.01

^d^*p *< 0.05

**Table 3:** Heritability estimates for selected discrete phenotypes retained in the final model

		Model test	*p*-Value
LNMXDK		POL vs. sporadic	1.37 × 10^-17c^
		EMF vs. POL	0.008^c^
		MIT vs. EMF	0.418
		XFX vs. EMF	0.500
final model:^a^	σ^2^_g_: 0.22(0.00^b^) + σ^2^_ef_: 0.32(0.00^b^) + σ^2^_em_: 0.42(0.00†)		
ttdt2		POL vs. sporadic	6.48 × 10^-12c^
		EMF vs. POL	0.000^c^
		MIT vs. EMF	0.136
		XFX vs. EMF	0.217
final model:^a^	σ^2^_g_: 0.28(0.00^b^) + σ^2^_ef_: 0.70(0.00^b^)+σ^2^_em_: 0.40(0.00†)		
LNCIGP		POL vs. sporadic	5.39 × 10^-14c^
		EMF vs. POL	0.059
		MIT vs. POL	0.037^d^
		XFX vs. MIT	0.500
final model:^a^	σ^2^_g_: 17.55(9.24) + σ^2^_e_: 333.79(0.29) + σ^2^_mt_: 55.06(2.46)		
ecb21		POL vs. sporadic	7.34 × 10^-35c^
		EMF vs. POL	0.002^b^
		MIT vs. EMF	0.012^d^
		XFX vs. MIT	0.234
final model:^a^	σ^2^_g_: 10.76(0.21) + σ^2^_ef_: 17.22(0.07) + σ^2^_em_: 10.50(0.07) + σ^2^_mt_: 3.24(0.21)		
ttth1		POL vs. sporadic	6.25 × 10^-11c^
		EMF vs. POL	0.149
		MIT vs. POL	0.030^d^
		XFX vs. MIT	0.500
final model:^a^	σ^2^_g_: 0.08(0.01) + σ^2^_e_: 0.28(0.00^b^) + σ^2^_mt_: 0.04(0.00^b^)		
ntth1		POL vs. sporadic	5.93 × 10^-9c^
		EMF vs. POL	0.010^d^
		MIT vs. EMF	0.060
		XFX vs. EMF	0.021^d^
final model:^a^	σ^2^_g_: 0.02(0.01) + σ^2^_ef_: 0.19(0.00^b^) + σ^2^_em_: 0.11(0.00^b^)+σ^2^_Xf_: 0.03(0.00^b^)		

^a^Estimates (SE) of parameters retained in the final model (the final estimate of h^2^_g _is given even when = 0). σ^2^_g_, autosomal additive genetic variance; σ^2^_mt_, additive genetic variance due to the mitochondrion; σ^2^_Xf_, X-linked additive genetic variance in females; σ^2^_ef_, random environmental variance in females; σ^2^_em_, random environmental variance in males.

^b^SEs reported as (0.00) are nonzero but <0.01.

^c^p < 0.01

^d^*p *< 0.05

**Table 4:** Nested models for traits LNCIGP and ntth1

Trait	Log-likelihood	σ^2^_g_	σ^2^_e_	σ^2^_ef_	σ^2^_em_	σ^2^_mt_	σ^2^_Xf_
LNCIGP							
A-polygenic	-1048.50	0.54	1.53	--	--	--	--
B-A+ diff σ^2^_e_	-1047.28	0.52	--^a^	1.66	1.40	--	--
C-all parameters	-1045.51	0.35	--	1.72	1.44	0.15	0.00
D-eq. σ^2^_e_	-1046.91	0.36	1.58	--	--	0.15	0.00
E-σ^2^_mt _= 0	-1047.24	0.49	--	1.67	1.38	--	0.03
F-σ^2^_xf _= 0	-1045.51	0.34	--	1.72	1.44	0.15	--
ntth1							
A-polygenic	224.36	0.07	0.15	--	--	--	--
B-A+ diff σ^2^_e_	227.06	0.07	--	0.17	0.13	--	--
C-all parameters	229.40	0.02	--	0.19	0.12	0.01	0.03
D-eq. σ^2^_e_	225.33	0.05	0.16	--	--	0.02	0.00
E-σ^2^_mt _= 0	229.14	0.02	--	0.19	0.11	--	0.03
F-σ^2^_xf _= 0	228.27	0.04	--	0.18	0.13	0.02	--
G-A + σ^2^_mt_	225.33	0.05	0.16	--	--	0.02	--
H-A + σ^2^_xf_	224.37	0.07	0.15	--	--	--	0.00

^a^--, parameter not estimated.

**Table 5:** Variance estimates for selected continuous phenotypes in the final model

	Estimate (SE) of each parameter retained in the final model^a^					
	
	POL		EMF		MIT	XFX
				
Trait	σ^2^_g_	σ^2^_e_	σ^2^_ef_	σ^2^_em_	σ^2^_mt_	σ^2^_Xf_
LNMXDK	0.22 (0.00)^b^	--^c^	0.32 (0.00)	0.42 (0.00)	--	--
ttd2	0.28 (0.00)	--	0.70 (0.00)	0.40 (0.00)	--	--
LNCIGP	17.55 (9.24)	333.79 (0.29)	--	--	55.06 (2.46)	--
ecb21	10.76 (0.21)	--	17.22 (0.07)	10.50 (0.07)	3.24 (0.21)	--
ttth1	0.08 (0.01)	0.28 (0.00)	--	--	0.04 (0.00)	--
ntth1	0.02 (0.01)	--	0.19 (0.00)	0.11 (0.00)	--	0.03 (0.00)

^a^σ^2^_g_, the autosomal additive genetic variance; σ^2^_mt_, the additive genetic variance due to the mitochondrion; and σ^2^_Xf_, the X-linked additive genetic variance in females; σ^2^_ef_, random environmental variances in females; σ^2^_em_, the random environmental variances in males.

^b^SEs reported as (0.00) are nonzero but <0.01.

^c^--, parameter not estimated.
